# Supplementary material for: Transcriptomic Analysis of Mating Responses in Bemisia tabaci MED Females
Source: Insects. 2020 May 14;11(5):308. doi: 10.3390/insects11050308 (PMC7290661; doi:10.3390/insects11050308)
Supplement: Supplementary file 1 [file insects-11-00308-s001.zip › Supplementary.docx]

Figure S1. Flow chart for sequencing and analysis


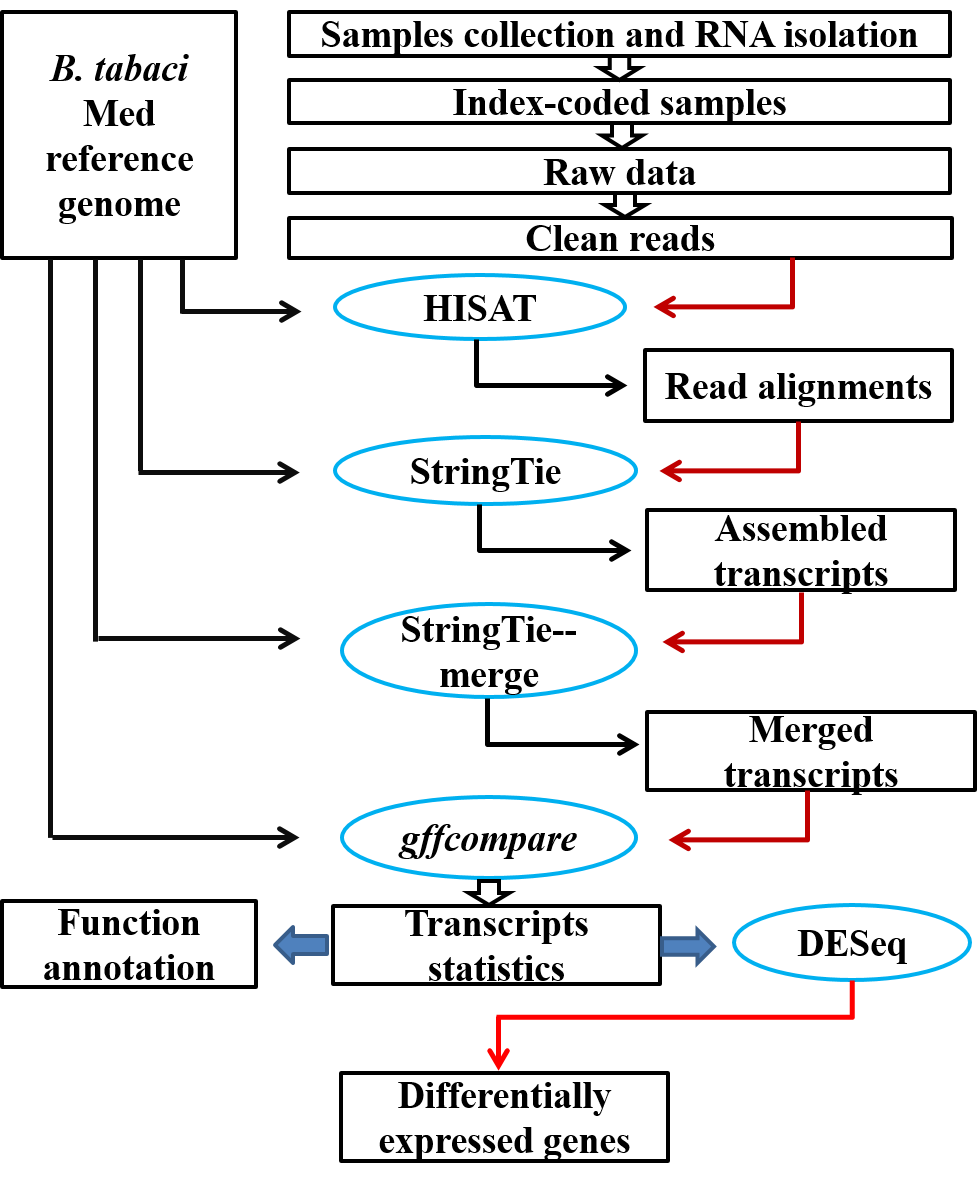


Figure S2. Distribution of all-annotated genes among the GO terms in the biological process, cellular component, and molecular function categories.
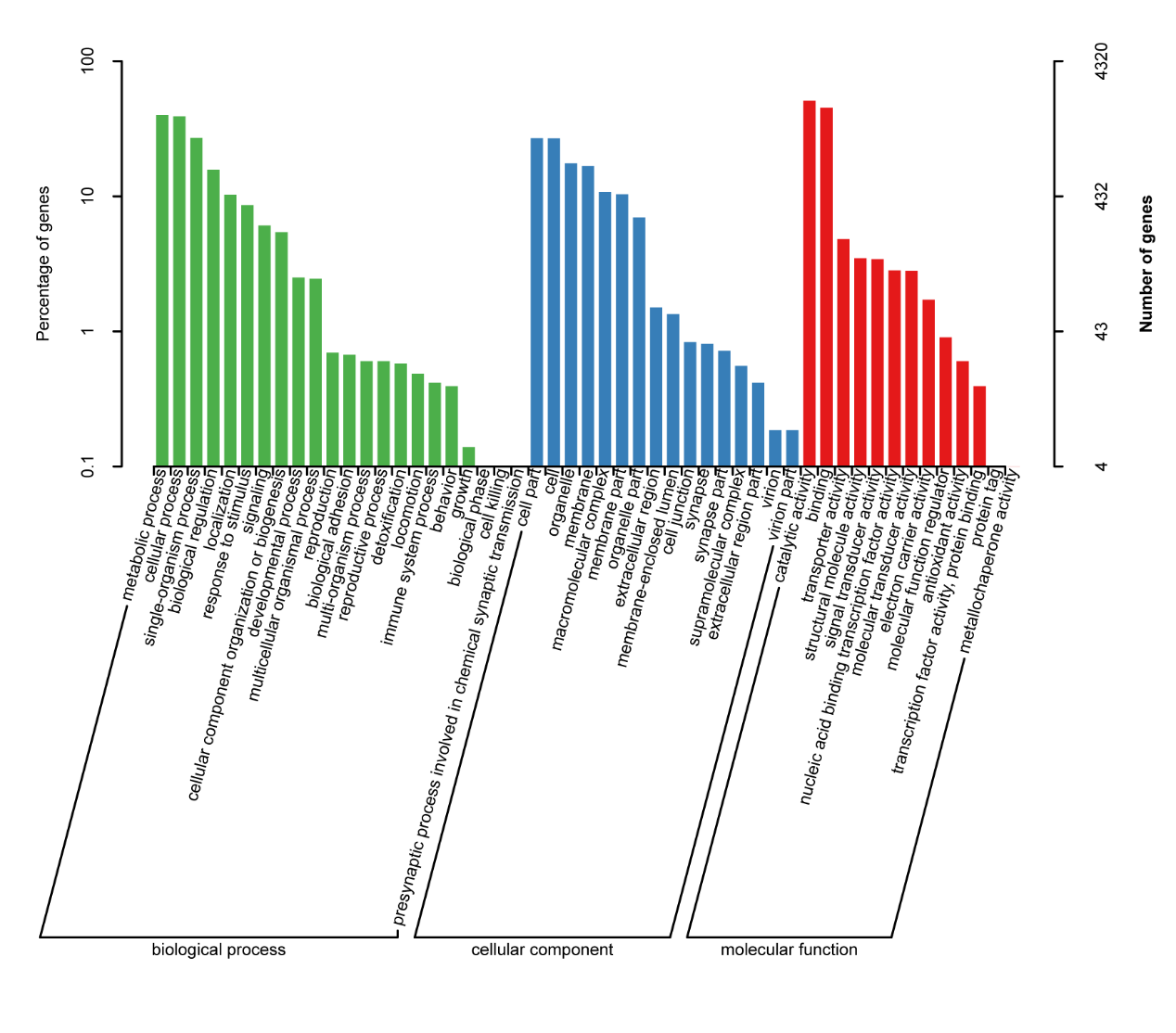


Figure S3. All-annotated genes among the KEGG classiﬁcations.


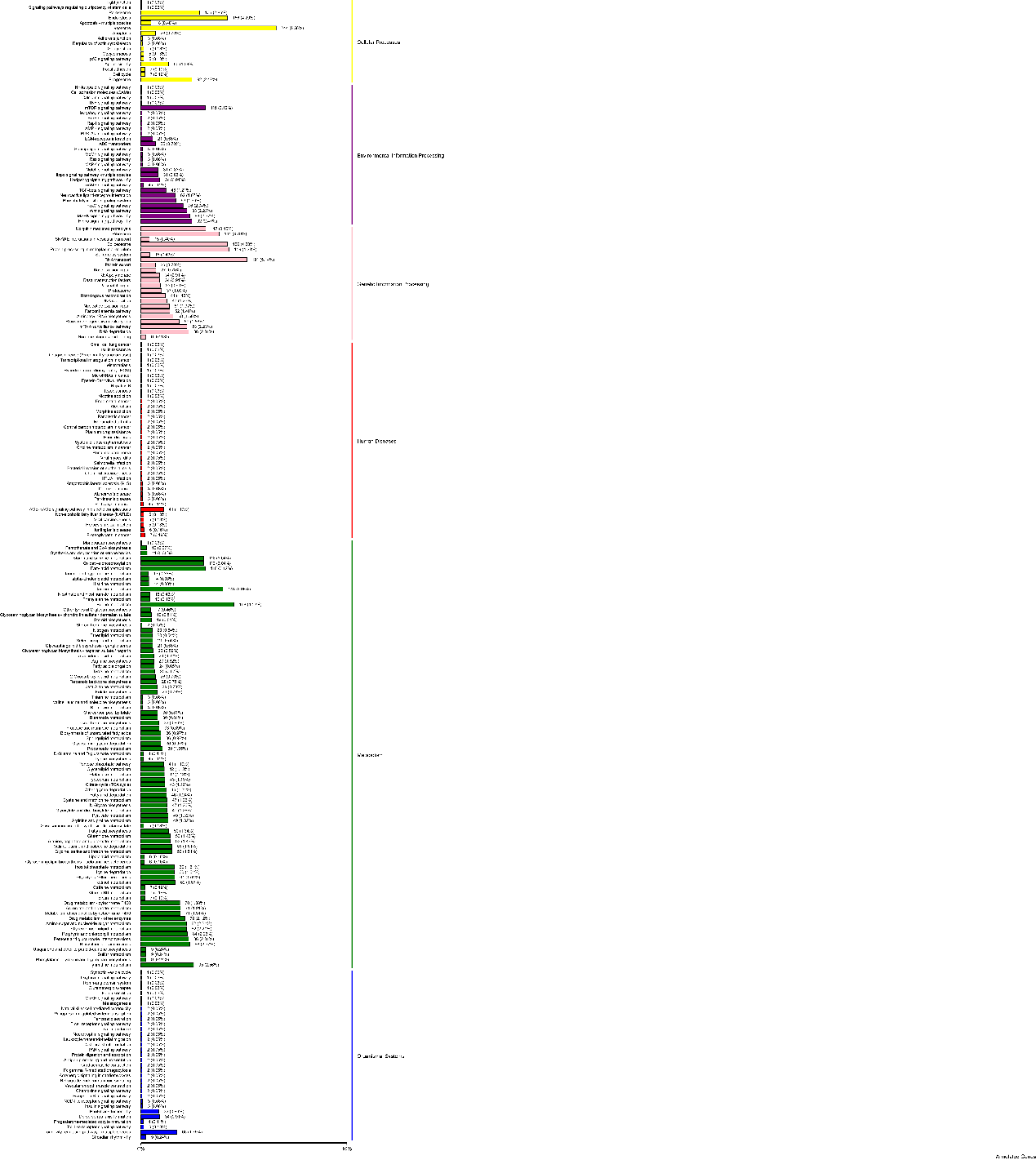


Figure S4. The PCA analysis samples.


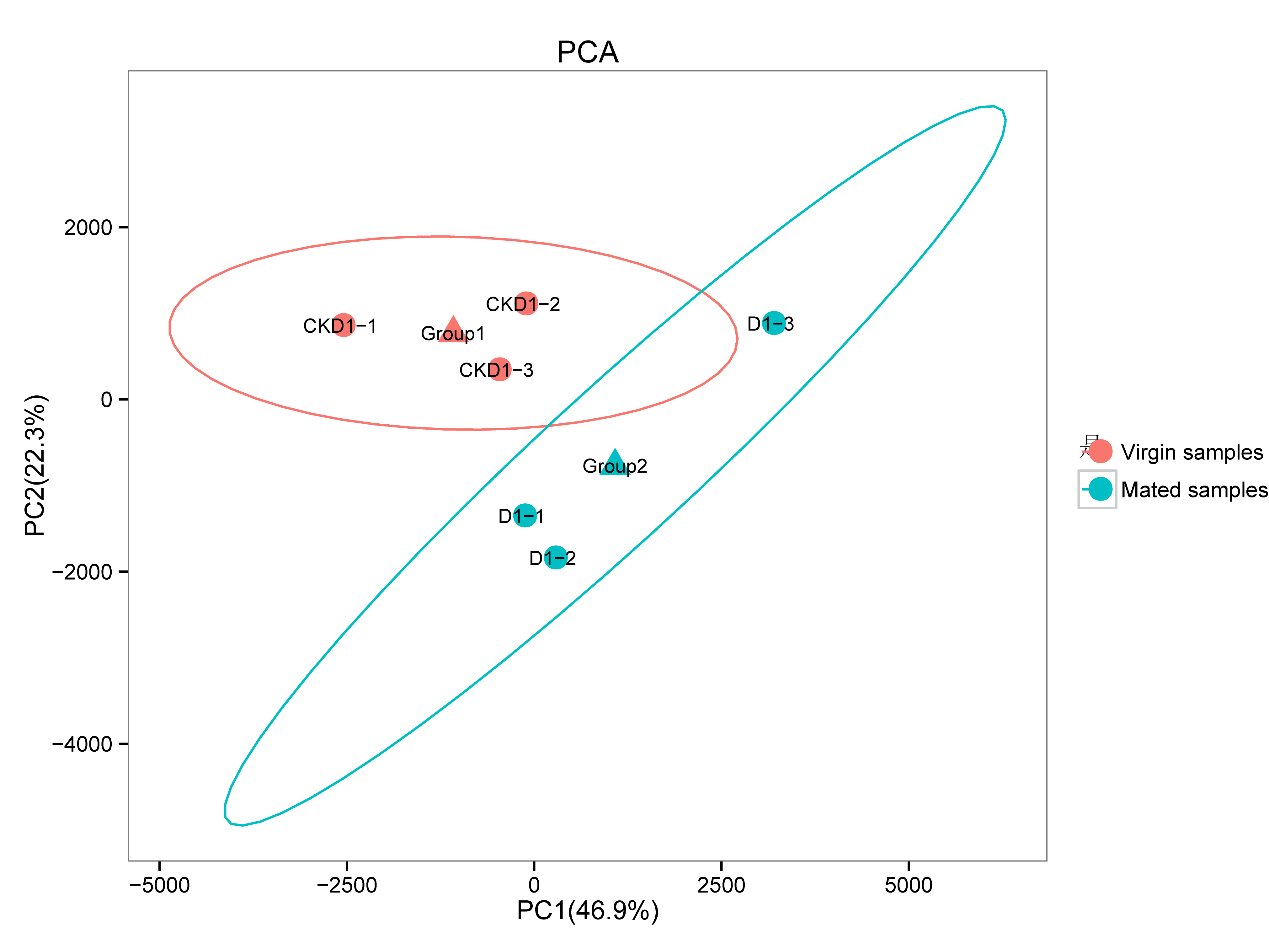


Figure S4. The PCA analysis of samples. Virgin samples: CKD1-1, CKD1-2, CKD1-3. Mated samples: D1-1, D1-2, D1-3.

Figure S5. Distribution of up- and down-regulated DEGs among the KEGG pathways.

**

**

Table S1. Primers used for qRT-PCR analysis.

| **Gene name** | **Primer name** | **Primer sequence (5′-3′)** | **TM(℃)** | **E** | **R^2^** |
| --- | --- | --- | --- | --- | --- |
| *DDAH-1* | DDAH-1-F | CCATGAACGTCTACAGCGAC | 60 | 105.25% | 0.9992 |
|  | DDAH-1-R | AAATCGACGGAGATGGGGTT |  |  |  |
| *ACC-1* | ACC-1-F | AGACGCAAAATCTCGAGTGC | 60 | 102.80% | 0.9973 |
|  | ACC-1-R | ATGACCGCTTTGCTCTTCAC |  |  |  |
| *ACC-2* | ACC-2-F | TGTGGTCTAATGAGCCTGGTTT | 60 | 105.96% | 0.9997 |
|  | ACC-2-R | CCATTTGATTGTGACGCAGTTC |  |  |  |
| *Cyp4g68* | Cyp4g68-F | ATAAGCCGTTTCTCGTGC | 60 | 100.98% | 0.9985 |
|  | Cyp4g68-R | AGTTGAGGAGATGCCTGGAC |  |  |  |
| *Cyp18a1* | Cyp18a1-F | TTTTCTCCACCAAAGTCGGCAA | 60 | 100.76% | 0.9989 |
|  | Cyp18a1-R | ATTTTCTTTGATTTTTCCACAGCCT |  |  |  |
| *UBR5* | UBR5-F | GATCAACAGCAGCAGTACGG | 60 | 101.37% | 0.9994 |
|  | UBR5-R | CGGCATCATCGGTAGTCTCT |  |  |  |
| *RNF123* | RNF123-F | AAACCCTCGCCACAAGTTTC | 60 | 103.93% | 0.9988 |
|  | RNF123-R | AGTGAAGACAAAGCGTGCTG |  |  |  |
| *UHRF1* | UHRF1-F | CGCCTTATTGTACTCGGTGC | 60 | 105.95% | 0.9997 |
|  | UHRF1-R | GCGCAGTACCATTCGTCAAT |  |  |  |
| *Tret1* | Tret1-F | GAGTCTCCCACATGGCTGAT | 60 | 104.66% | 0.9983 |
|  | Tret1-R | CTTAGGCCAGCAGAGTTCCT |  |  |  |
| *Tret1-2* | Tret1-2-F | CTAACGTGGCCAACAGTAGC | 60 | 105.82% | 0.9995 |
|  | Tret1-2-R | TCCTAAGCGTCTTTGAGCCA |  |  |  |
| *TLK2* | TLK2-F | CGGAAACAGGACGGAAAAGG | 59 | 100.00% | 0.9958 |
|  | TLK2-R | CACGATAAGCCAGACACAGC |  |  |  |
| *SBK1* | SBK1-F | GCAAGAGGGTGGCTCAACA | 59 | 94.59% | 0.9952 |
|  | SBK1-R | TCGCCGAAGTCGCACAG |  |  |  |
| *phtf* | phtf-F | CTCTGAAACCGCTGGCTGAA | 60 | 102.96% | 0.9995 |
|  | phtf-R | GCAAACGGGCTATCATTATCAA |  |  |  |
| *Hsp68* | Hsp68-F | CCGCCTAGTCAACCATTTCG | 60 | 96.90% | 0.9982 |
|  | Hsp68-R | CAGCAGAGCGTCGATTTCAA |  |  |  |
| *Hsf* | Hsf-F | GACAGTTGAATATGTATGGCTTCC | 59 | 103.83% | 0.9996 |
|  | Hsf-R | AAGGTATGGATGACCACGGATG |  |  |  |
| *RPL29* | RPL29-F | TCGGAAAATTACCGTGAG | 60 | Li et al., 2013 | |
|  | RPL29-R | GAACTTGTGATCTACTCCTCTCGTG |  |  |  |

E: amplification efficiency. R^2^: correlation coefficient.

Table S2. Statistics of sequencing analysis.

| **Sample** | **Clean reads** | **Clean bases** | **Total Reads** | **Q30 percentage** | **GC Content** | **Total Mapping Reads Percentage** |
| --- | --- | --- | --- | --- | --- | --- |
| CKD1-1 | 20,945,578 | 6,263,630,700 | 41,891,156 | 93.24% | 39.16% | 75.51% |
| CKD1-2 | 28,167,920 | 8,410,792,294 | 56,335,840 | 93.41% | 41.17% | 80.23% |
| CKD1-3 | 20,664,597 | 6,176,631,750 | 41,329,194 | 92.27% | 39.47% | 78.29% |
| D1-1 | 25,643,890 | 7,647,391,432 | 51,287,780 | 93.40% | 40.05% | 81.92% |
| D1-2 | 21,532,138 | 6,429,892,448 | 43,064,276 | 93.00% | 39.89% | 81.33% |
| D1-3 | 20,724,777 | 6,175,983,568 | 41,449,554 | 93.65% | 43.90% | 83.52% |

Table S3. Statistics of sequencing results.

| Databases | Total | COG | GO | KEGG | KOG | NR | Pfam | Swiss-Prot | eggNOG |
| --- | --- | --- | --- | --- | --- | --- | --- | --- | --- |
| All transcripts | 25,594 | ̶ | ̶ | ̶ | ̶ | ̶ | ̶ | ̶ | ̶ |
| All-annotated | 20,912 | 7,146 | 4,320 | 8,008 | 11,381 | 20,474 | 15,009 | 10,554 | 17,238 |
| Novel genes | 4846 | ̶ | ̶ | ̶ | ̶ | ̶ | ̶ | ̶ | ̶ |
| Novel genes-annotated | 2,510 | 296 | 555 | 667 | 987 | 2,491 | 1,058 | 820 | 1,452 |
| ≤300bp | ̶ | 74 | 121 | 151 | 208 | 638 | 227 | 198 | 375 |
| 300bp-1000bp | ̶ | 2,126 | 1,690 | 2,548 | 3,627 | 7,529 | 4,870 | 3,231 | 5,977 |
| ≥1000bp | ̶ | 4,946 | 2,509 | 5,309 | 7,546 | 12,307 | 9,912 | 7,125 | 10,886 |

Table S8. GO terms enrichment of top 20.

| **GO Term** | **GO ID** | **DEGs numbers** | **KS** |
| --- | --- | --- | --- |
| nucleus | GO:0005634 | 6 | 6.90E-06 |
| transcription factor activity, sequence-specific DNA binding | GO:0003700 | 3 | 0.00021 |
| sequence-specific DNA binding | GO:0043565 | 2 | 0.00172 |
| DNA binding | GO:0003677 | 6 | 0.00221 |
| intracellular part | GO:0044424 | 13 | 0.0023 |
| oxidoreductase activity, acting on CH-OH group of donors | GO:0016614 | 2 | 0.00489 |
| transcription, DNA-templated | GO:0006351 | 5 | 0.0124 |
| RNA biosynthetic process | GO:0032774 | 5 | 0.0211 |
| single-organism metabolic process | GO:0044710 | 12 | 0.0234 |
| regulation of nucleobase-containing compound metabolic process | GO:0019219 | 3 | 0.0262 |
| molecular transducer activity | GO:0060089 | 2 | 0.02714 |
| signal transducer activity | GO:0004871 | 2 | 0.02714 |
| signal transduction | GO:0007165 | 6 | 0.0289 |
| regulation of transcription, DNA-templated | GO:0006355 | 3 | 0.0311 |
| regulation of nitrogen compound metabolic process | GO:0051171 | 3 | 0.0318 |
| protein complex | GO:0043234 | 6 | 0.0355 |
| regulation of RNA metabolic process | GO:0051252 | 3 | 0.0356 |
| regulation of cellular macromolecule biosynthetic process | GO:2000112 | 3 | 0.0367 |
| response to stimulus | GO:0050896 | 11 | 0.0408 |
| small GTPase mediated signal transduction | GO:0007264 | 2 | 0.0411 |

“KS”: Kolmogorov–Smirnov，The smaller the KS value, the more significant the enrichment.

Table S11. Functional annotation of selected genes.

| **Sequence ID*** | **Gene name** | **Annotation** |
| --- | --- | --- |
| BTA020297.1.gene | *DDAH-1* | N-Dimethylarginine dimethylaminohydrolase [*Curtobacterium sp.* 9128] |
| Bemisia_tabaci_newGene_5452 | *ACC-1* | PREDICTED: acetyl-CoA carboxylase isoform X1 [*Bemisia tabaci*] |
| BTA006415.1.gene | *ACC-2* | PREDICTED: acetyl-CoA carboxylase isoform X1 [*Bemisia tabaci*] |
| BTA022151.1.gene | *Cyp4g68* | cytochrome P450 4g68 [*Bemisia tabaci*] |
| BTA020299.1.gene | *Cyp18a1* | PREDICTED: cytochrome P450 18a1 [*Bemisia tabaci*] |
| BTA020192.1.gene | *UBR5* | PREDICTED: E3 ubiquitin-protein ligase UBR5 isoform X4 [*Bemisia tabaci*] |
| BTA027639.1.gene | *RNF123* | PREDICTED: E3 ubiquitin-protein ligase RNF123 [*Bemisia tabaci*] |
| BTA013219.1.gene | *UHRF1* | PREDICTED: E3 ubiquitin-protein ligase UHRF1-like [*Bemisia tabaci*] |
| BTA013778.1.gene | *Tret1* | PREDICTED: facilitated trehalose transporter Tret1-like [*Bemisia tabaci*] |
| BTA013908.1.gene | *Tret1-2* | PREDICTED: facilitated trehalose transporter Tret1-like [*Bemisia tabaci*] |
| BTA014562.1.gene | *TLK2* | PREDICTED: serine/threonine-protein kinase tousled-like 2 isoform X1 [*Bemisia tabaci*] |
| BTA005900.1.gene | *SBK1* | PREDICTED: serine/threonine-protein kinase SBK1 [*Bemisia tabaci*] |
| BTA000065.1.gene | *phtf* | PREDICTED: putative homeodomain transcription factor isoform X2 [*Bemisia tabaci*] |
| BTA011604.1.gene | *Hsp68* | heat shock protein 70 [*Bemisia tabaci*] |
| BTA008456.1.gene | *Hsf* | PREDICTED: heat shock factor protein isoform X2 [*Bemisia tabaci*] |

“*”: Refered from the ref (Xie W, Chen C, Yang Z, et al. Genome sequencing of the sweetpotato whitefly *Bemisia tabaci* MED/Q[J]. GigaScience, 2017, 6(5): gix018).

Table S12. Correlations between qRT-PCR and RNA-seq analyses.

| **Gene name** | **R** | **P** |
| --- | --- | --- |
| *DDAH-1* | 0.937 | 0.006 |
| *Cyp4g68* | 0.862 | 0.025 |
| *Cyp18a1* | 0.949 | 0.004 |
| *UBR5* | 0.902 | 0.014 |
| *RNF123* | 0.818 | 0.047 |
| *UHRF1* | -0.401 | -0.431 |
| *Hsp68* | 0.864 | 0.027 |
| *Hsf* | 0.991 | 0 |
| *ACC-1* | 0.832 | 0.04 |
| *ACC-2* | 0.907 | 0.012 |
| *Tret1* | 0.955 | 0.003 |
| *Tret1-2* | 0.96 | 0.002 |
| *phtf* | 0.866 | 0.026 |
| *TLK2* | 0.827 | 0.042 |
| *SBK1* | 0.936 | 0.006 |

R: correlation coefficient; P < 0.05: Significant correlation between qRT-PCR and RNA-seq.
