# Supplementary figures and images for: Transcriptomic Analysis of Mating Responses in Bemisia tabaci MED Females
Source: Insects. 2020 May 14;11(5):308. doi: 10.3390/insects11050308 (PMC7290661; doi:10.3390/insects11050308)

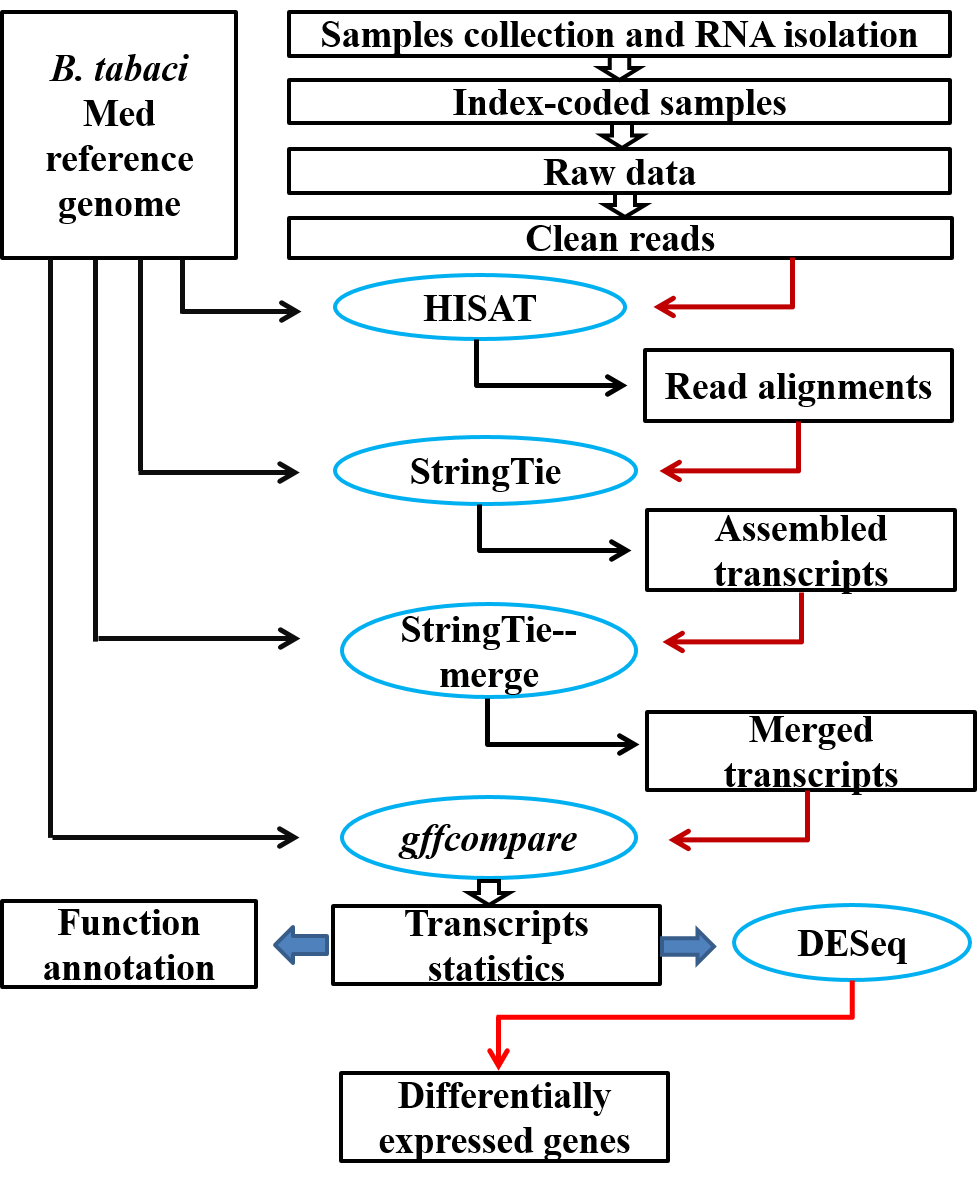

Supplement: Supplementary file 1 [file insects-11-00308-s001.zip › Figure S1.tif]

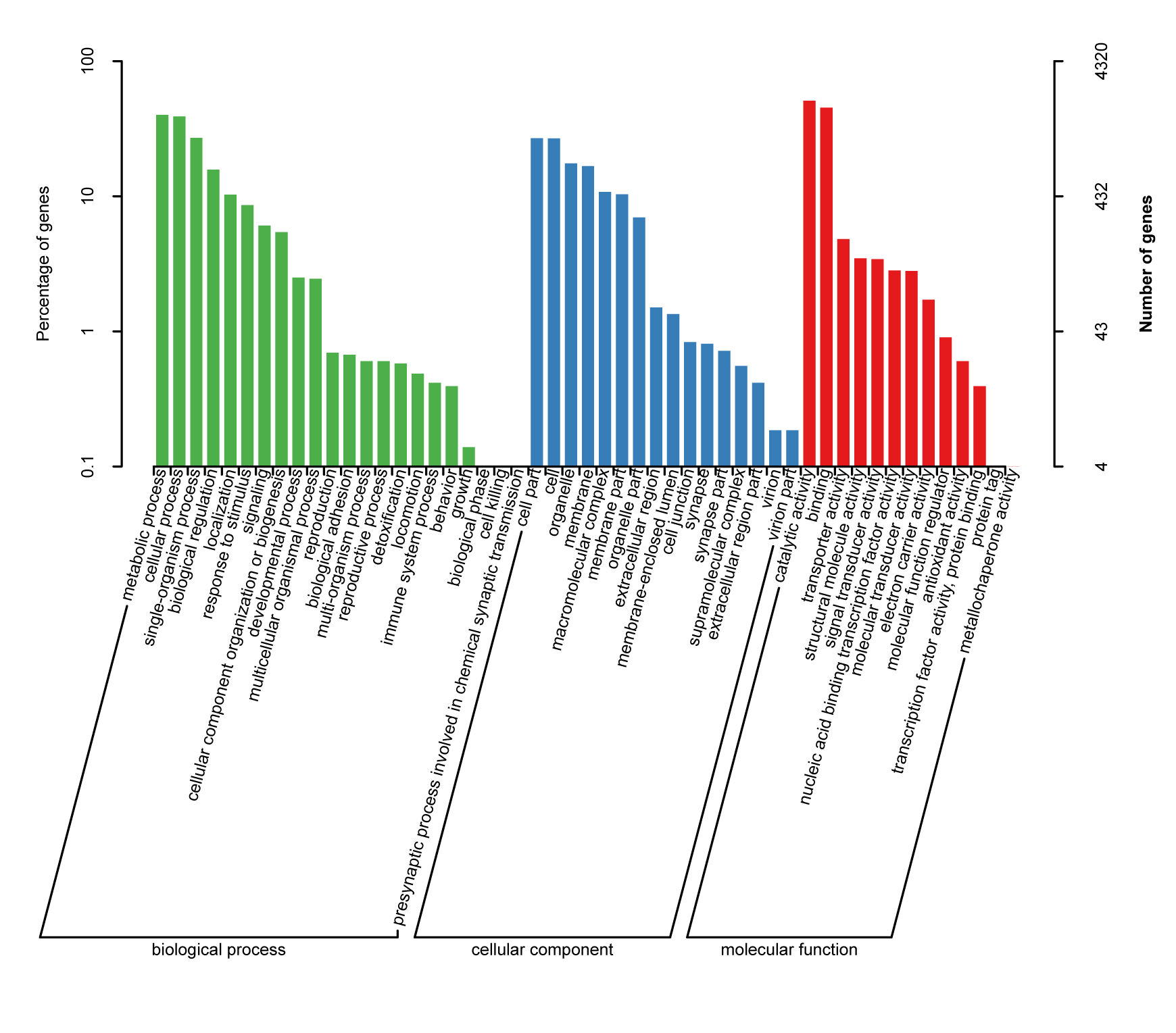

Supplement: Supplementary file 1 [file insects-11-00308-s001.zip › Figure S2.tif]

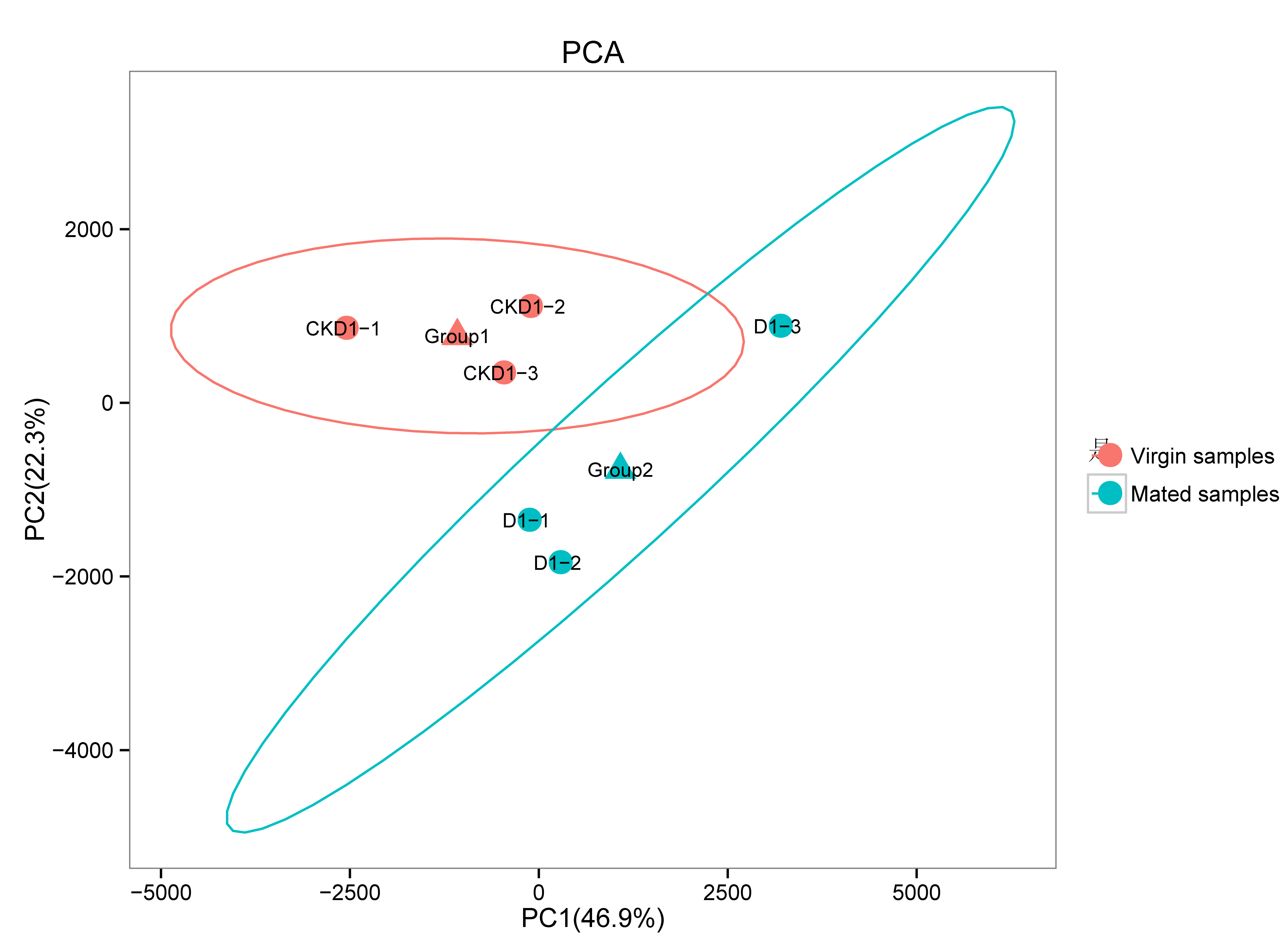

Supplement: Supplementary file 1 [file insects-11-00308-s001.zip › Figure S4.tif]
